# Beyond Online Course Evaluations: Student-Led Focus Groups for Curricular Feedback

**DOI:** 10.5334/pme.1786

**Published:** 2026-09-09

**Authors:** David Rekhtman, Jessica Campanile, Alex F. Nisbet, Abigail Kohler, Jennifer Hong, Ish Sethi, Suzanne Rose, C. Jessica Dine

**Affiliations:** 1 Perelman School of Medicine, University of Pennsylvania, Philadelphia, PA, US

## Abstract

**Purpose:**

Medical student curricular evaluation is both beneficial and required. Most medical schools rely on online questionnaires for evaluations, but these are often overlooked or completed haphazardly. We describe the implementation of focus groups as a supplemental form of curricular assessment and evaluate students’ and faculty members’ attitudes towards this novel approach.

**Method:**

Ten randomly selected students were assigned to eight focus groups regarding three different preclerkship courses. Following the completion of focus groups, interviews were conducted with students who participated in them, course directors who received the focus group reports, and administrators who oversaw the preclerkship curriculum and the implementation of the focus group program. The semi-structured interviews were then analyzed using inductive thematic analysis, with initial codes generated from the data and subsequent interpretation informed by prior literature on feedback relationships and curricular program evaluation.

**Results:**

Interviews were conducted with 17 students, 5 faculty members, and 3 administrators. Participants highlighted key themes related to trust, engagement, collaboration, and the learning environment. Students described the focus groups as a safe space and said they enjoyed thinking critically about improvements. Faculty perceived the thematic content of focus group reports to be comparable to course evaluation summaries from established online forms despite concerns for a smaller sample size.

**Conclusions:**

Focus groups are a novel supplemental tool that can complement existing evaluation mechanisms. Engaging students through focus groups can improve feedback and advance medical school curricula, resulting in a more responsive and adaptive approach to education.

## Introduction

Medical school curricula are constantly evolving in response to advancements in science, patient care, and teaching modalities. Thorough evaluation of a school’s curriculum allows for improvements in content delivery. Although various stakeholders play important roles in driving curricular reform, feedback from medical students is paramount. The Liaison Committee on Medical Education, the accrediting body for undergraduate medical education programs in the United States, requires all accredited medical schools to collect outcome data on their courses (standard 8.4) as well as develop a formal process for medical students to evaluate courses, clinical experiences, and faculty (standard 8.5) [1].

Today, the majority of medical schools ask students to complete online questionnaires to evaluate courses and educational activities [2,3]. Although there is room for additional free-text responses, the standardization of the form limits the specificity of the feedback [4]. Furthermore, due to the sheer number of feedback forms, students often experience feedback fatigue, leading to disengaged or perfunctory responses [5]. The collected ratings may be invalid, and the free-text comments are often sparse and disjointed [4]. As a result, the current system does not incentivize students to reflect on the material and its delivery, thereby inhibiting thoughtful suggestions and minimizing the utility of current methods for informing curricular advancement.

Medical schools have recognized the need to develop alternative mechanisms for acquiring student feedback. Focus groups have become a popular method for qualitative data collection [4,6,7]. Originally intended for marketing research, focus groups have been used in many educational settings to elicit a group’s perspective on a specific course or learning environment, such as a preclerkship course [4,8]. These sessions provide students an opportunity to reflect on their experiences, discuss both positive and negative components of a course, and identify actionable avenues for improvement [8]. Importantly, the qualitative narrative provides nuance and context, as members of a focus group can debate their ideas and compare various perspectives. Prior literature suggests that structured, dialogic feedback processes may foster trust, engagement, and a sense of shared purpose between learners and educators [9]. This dynamic may enhance student engagement in the feedback process and increase the likelihood of meaningful curricular change. However, focus groups are more time-intensive for students, and the reported outcomes may be subject to bias and groupthink [10].

In this study, we describe the implementation of student-led focus groups for preclerkship curricular feedback at our institution. Additionally, we assessed the students’ experiences in the focus group, the course director’s perception of the recommendations developed by the group, and the administrative viewpoint on the initiative’s feasibility.

## Methods

### Study Context: Student-Led Focus Groups for Curricular Feedback

As context for this study, our institution implemented a student-led focus group program to supplement existing curricular feedback mechanisms. The primary method of curricular evaluation at our institution is an online survey platform (OASIS, Schilling Consulting LLC, Madison, Wisconsin), through which students complete brief Likert-scale evaluations of individual educational activities and end-of-course summaries, with optional free-text comments. Additionally, elected student “Course Representatives” meet regularly with course leadership to provide informal feedback.

To complement these mechanisms, a pilot program of student-led focus groups was introduced in the preclerkship curriculum. Ten randomly selected students were invited to participate in each of eight focus groups across three courses. Trained student facilitators led one-hour sessions every other week throughout the preclerkship courses in which they were piloted. After the focus groups for a course concluded, the student facilitators developed a summary report for the course directors and the Program Evaluation Committee (PEC).

During Spring 2024, 42 of 80 invited students (52.50%) participated in the pilot evaluation project, with focus group sizes ranging from 2 to 7 (median 5.5). With regard to demographics, 52% of participants were female, and the median age was 24. Races included Asian (35.71%), Black (9.52%), Hispanic (11.91%), White (28.57%), and other (14.29%), which is fairly representative of the class as a whole. Facilitators generated two- to four-page summaries for each group, which were distributed within two weeks. During the same period, response rates for status quo online course evaluations were 20% (n = 33), 47% (n = 78), and 44% (n = 72) for the three participating courses.

### Design: Exit-Interviews

To evaluate the pilot evaluation project, we conducted a descriptive qualitative study using inductive thematic analysis [11]. Semi-structured interviews of students and faculty were conducted between October and December 2024 by one of the authors (DR). Interview transcripts were coded without predefined categories, allowing themes to emerge directly from participants’ narratives. The analysis was iterative and reflexive, with ongoing discussion among the research team to refine themes and sub-themes. After themes were developed, we examined how they related to existing literature on feedback relationships and learning environments, including educational alliance theory [9,12]. The literature was used interpretively to contextualize findings rather than to guide data collection or initial coding. The research team, composed of medical students and faculty, brought diverse perspectives to the analysis, with the lead investigator (CJD) contributing expertise in evaluating curricular feedback and qualitative research.

### Participants and Setting

All students who had participated in preclerkship focus groups in 2024 were invited to interview via email. Student performance in the course was unknown at that time. All course directors, as well as the Chair of PEC, the Associate Dean of UME Science and Discovery Curriculum, and the Associate Dean for Student Success and Professional Development, were also invited to interview. Course directors are intimately involved in student education as they design courses, organize lectures, form small groups, and write exams. The Chair of PEC is responsible for leading a committee that reviews all course evaluations that are collected from OASIS. The Chair was also a course director for one of the preclerkship courses. The Associate Deans met with Course Representatives weekly to discuss immediate feedback and are also voting members on PEC.

Interviews were conducted on Zoom (Zoom Video Communications, San Jose, California), allowing for audio recording and subsequent transcription (Rev, Austin, Texas). This study was reviewed by the Institutional Review Board at the University of Pennsylvania and was determined to be exempt from full ethical review (Protocol #854912).

### Data Collection

One researcher (DR) conducted all semi-structured interviews with students and faculty about their experience participating in a focus group and receiving a focus group report, respectively. The research team designed interview questions based on prior participant feedback, with a focus on psychological safety, engagement, and representation (Supplementary Table 1) (DR, CJD). Additionally, interviewees were asked to provide suggestions for improving both the focus group structure and report content. A key component of the interviews examined how the focus group reports functioned within existing feedback mechanisms, including OASIS and Course Representatives. Interviews were continued until thematic saturation was reached, defined as the point at which no new codes or themes emerged during iterative review of consecutive transcripts [13].

### Data Analysis

This analysis was conducted within a pragmatic qualitative paradigm informed by a constructivist understanding that participants’ experiences and perceptions are context-dependent and co-constructed through their interactions with one another and the interview process [14]. Accordingly, our goal was to generate an interpretive understanding of stakeholder perspectives rather than identify an objective account of the phenomenon. We combined an inductive thematic approach with analytic strategies intended to enhance transparency and credibility in an applied educational context [15]. Two members of the research team (DR and CJD) used inductive thematic analysis to generate initial codes directly from the data [11]. Codes were iteratively compared and refined through team discussion with additional co-authors (JC and AN), leading to the development of higher-order themes (Supplemental Table 2). Each transcript was assessed by two team members using NVivo14 (QSR International Inc., Burlington, Massachusetts). Discrepancies were discussed until consensus was achieved, with a third team member – one who had not previously assessed the transcript – providing adjudication as needed (DR, JC, or AN). Reporting the relative emphasis of themes and using multiple coders were pragmatic strategies to support analytic transparency and consistency rather than attempts to quantify findings or establish objective truth.

The research team included medical students and faculty members with direct experience in curricular evaluation and a vested interest in improving educational feedback processes. We recognize that these positions may have influenced data interpretation, particularly regarding the perceived value and feasibility of focus groups. To mitigate this, we used inductive coding without predefined themes, held regular team discussions to challenge assumptions, and included analysts with different institutional roles. Additionally, interview questions were designed to elicit both positive and critical perspectives, and discrepant views were actively discussed during analysis.

## Results

**Figure 1:**
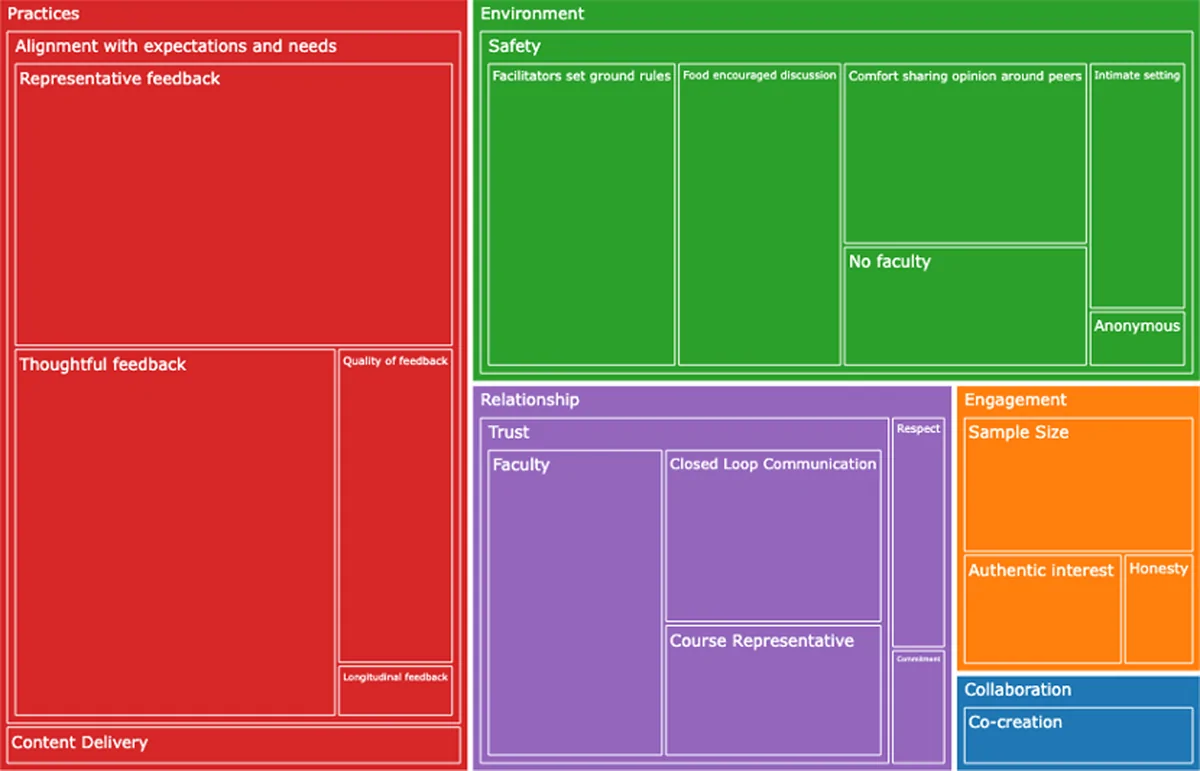
**Student Experience**. Tree map in which the size of the box corresponds to the proportional frequency of a specific code among transcripts obtained from interviews with students. This data is also presented in tabulated form in Table 1.

**Figure 2:**
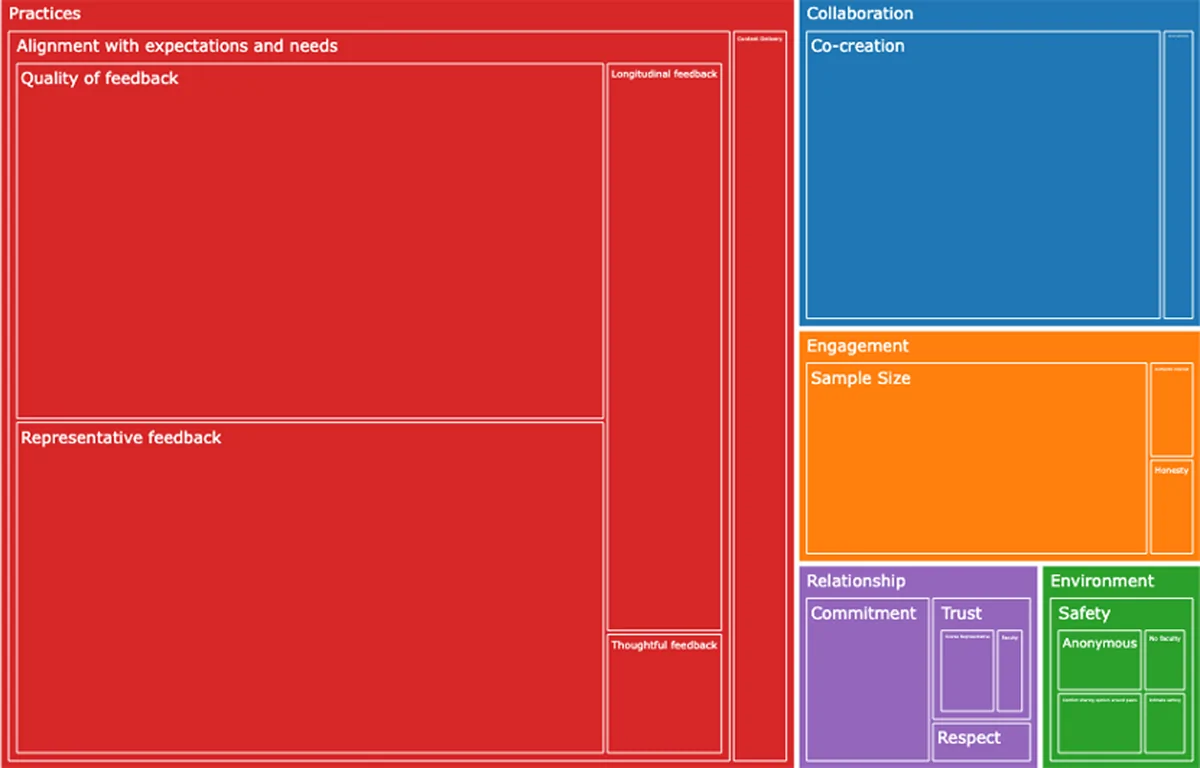
**Faculty Experience**. Tree map in which the size of the box corresponds to the proportional frequency of a specific code among transcripts obtained from interviews with course directors and administrators. This data is also presented in tabulated form in Table 1.

Seventeen students, five course directors, and three administrators participated in the exit interviews. Of the student participants, 53% were female, the median age was 24, and the racial distribution was similar to that of the focus group cohort. Performance in the course was similar when comparing those who were invited to the focus groups, participated in the focus groups, and participated in the exit interviews. Inductive thematic analysis yielded five main themes from participant narratives: practices, engagement, collaboration, relationship, and environment (Table 1; Supplemental Table 2). Although both students and faculty discussed the same themes, they varied in frequency and emphasis (Figures 1 and 2).

**Table 1:** Themes identified following exit-interviews with students and faculty.


CODE	# OF STUDENT REFERENCES	# OF FACULTY REFERENCES	PERCENT TOTAL

Collaboration	1	12	**2.42**

Co-creation	1	11	2.23

Shared Responsibility	0	1	0.19

Engagement	29	21	**9.29**

Authentic interest	9	1	1.86

Honesty	4	1	0.93

Sample Size	16	19	6.51

Environment	115	6	**22.49**

Safety	115	6	22.49

Anonymous	3	2	0.93

Peers	24	2	4.83

Facilitators	31	0	5.76

Food	27	0	5.02

Intimate Setting	13	1	2.60

No faculty	16	1	3.16

Practices	148	116	**49.07**

Alignment with expectations and needs	140	108	46.10

Longitudinal feedback	3	14	3.16

Quality of feedback	18	44	11.52

Representative feedback	61	41	18.96

Focus Groups	48	22	13.01

OASIS	12	18	5.58

Thoughtful engagement with feedback	58	3	11.34

Student engagement with OASIS	28	1	5.39

Significant time or work burden	17	1	3.35

Student participation in OASIS	13	1	2.60

Student engagement in focus groups	17	1	3.35

Content delivery	8	8	2.97

Relationship	80	10	**16.73**

Commitment	3	5	1.49

Respect	6	1	1.30

Trust	71	4	13.94

Closed-loop communication	21	0	3.90

Course Representatives	16	2	3.35

Faculty	30	1	5.76


### The Student Experience

#### Practices

The most common theme discussed by students was “practices”. Students repeatedly expressed frustration with the burden of completing the online evaluation forms for each lecture and small group activity in the preclerkship curriculum:

*“I don’t really do the surveys for the course evals because there’s just way too many of them.” (Student 8)*

*“When I’m filling out the surveys, I’m not even thinking; I’m just doing it so I don’t get email reminders again.” (Student 3)*

Focus groups offered students a space for thoughtful reflection and discussion without adding significant time or administrative burden. Peer opinions often sparked new ideas through agreement or disagreement. Students found the focus groups productive and manageable, describing them as a structured but reasonable obligation within the curriculum:

*“It was nice… [giving feedback] in a focus group, in a group setting, hearing what other people have to say, and then allowing that to trigger thoughts in my mind so that I could speak on that. So just having it be an open discussion was really helpful.” (Student 9)*

A few students acknowledged the advantages of the online survey that could not be replicated in a focus group. For one, it allowed for significantly more granular detail, as it evaluated each lecture and lecturer independently, while a focus group discussed the highlights over the course of two weeks. Additionally, the online forms were fully anonymous and created a private space for students to provide feedback who may not have felt comfortable sharing their opinions with the group.

Students recognized that the feedback produced through either forum must be representative to be useful. Those who participated in the focus group felt that the common sentiments among their classmates were reflected in the discussion. Some mentioned that they were representing not only their own views but also those of their peers when they participated in the focus group. Students noted that they felt comfortable enough to disagree with their peers and provide an alternative perspective. Notably, students thought that an in-person discussion would allow the narrative to “regress to the mean” rather than the polarized viewpoints that may be submitted using an anonymous online form.

#### Environment

Students also emphasized the importance of the environment being a safe space to ensure that feedback is representative and honest. They pointed to a few specific design elements that increased their willingness to share their thoughts. The most referenced component was the student facilitators, known members of their class, who set the tone for the focus group as a nonjudgmental space. They expressed empathy without bias towards all participants and encouraged discussion through open-ended questions. Students felt comfortable sharing with their peers, especially since it was a small, intimate setting of fewer than 10 students.

*“I feel like people weren’t afraid to share a different perspective. And I know I didn’t feel like it would be frowned upon if I shared an opinion that the group didn’t have at that one particular point because we are all peers in the focus group. I think it was a good environment that was nonjudgmental.” (Student 13)*

To some, the most important aspect was the free food, which served as an incentive and created a relaxed atmosphere where students could discuss topics more casually over a meal. The lack of faculty or leadership was an additional theme repeatedly noted among students, as it allowed for more relaxed and honest conversations, which they perceived as anonymous.

#### Relationship

Students frequently discussed trust as a central aspect of their relationships with both the facilitators and the faculty. Overall, students trusted the Course Representatives to represent their views in the report, but some noted that it would have been helpful to view the reports prior to submission to the course directors to ensure their opinions had been properly represented.

Student participants also discussed their trust in the faculty’s commitment to improving courses based on feedback. Participants referenced an institutional culture of seeking and responding to feedback through various avenues. Despite the novel format of the focus groups, students believed that faculty would take the reports seriously and act on the feedback. The focus groups were highlighted for providing greater context to feedback than isolated comments submitted through other platforms, potentially fostering greater responsiveness from faculty. Some students expressed a desire for more closed-loop communication from the faculty to highlight the changes made in response to submitted feedback.

*“I think course directors have always been receptive to feedback.” (Student 13)*

*“Sometimes I feel like if they understand the complaints, then they’ll act on them. If they don’t understand the complaints, then they won’t.” (Student 16)*

#### Engagement

Students repeatedly spoke highly of their focus group and said they enjoyed the sessions. Students appreciated the time spent and recognized the value in the initiative.

*“I personally don’t very often take the time to fill out the [feedback] forms, but when I was invited to the focus group, I showed up for that.” (Student 16)*

However, students recognized that not all students were invested in this feedback mechanism. Many students chose not to attend the focus groups, resulting in some sessions having very few participants. This not only reduced the representativeness of the feedback but also indicated that some students were not committed to the curricular assessment process being offered.

*“…the focus group I participated in was rather sparse, so I think it can get some opinions, but definitely not all because student experiences are so vastly different in courses.” (Student 17)*

*“Perhaps you don’t have students that come and then you end up having four or five people there or even less, which I think then it’s definitely not representative.” (Student 14)*

### The Faculty Perspective

#### Practices

Faculty expected the feedback to be specific and actionable. Repeatedly, participants noted that merely identifying issues – though important – was not as helpful since many other feedback avenues could highlight components of a course that needed to be improved. The value of a focus group was that it took it a step further by considering possible solutions. Focus group reports were not consistently able to provide the level of detail that was desired by faculty.

*“I was looking for more granularity.” (Administrator 1)*

*“I thought that I could tell that there were attempts to be very specific, which is really helpful as in feedback, being specific and actionable is important.” (Course Director 1)*

From the perspective of administrators who review the course on an annual basis, these reports were said to be beneficial. They provided a succinct narrative describing the course components that worked well and those that needed improvement. The context was especially valuable for reviewers who did not have intimate knowledge of the course design or content. However, the current review system relies on quantitative data, which are easily produced by online forms; summary statistics for lectures, small groups, and other components of the course are generated by the software to guide course initiatives and evaluations. This was not provided through entirely qualitative focus groups. Both methods of curricular assessment allowed for longitudinal reviews since changes could be noted from year to year. However, the focus groups also allowed for assessments at various points within a course (i.e., before and after the final exam) to capture students’ progress.

*“When we’re asked to review courses where many of us don’t have any intimate knowledge about it, I think it’s leaps and bounds better because otherwise, when you’re filtering through those OASIS comments … you are at the mercy of how thorough those OASIS reviews are….” (Administrator 3)*

*“A focus group obviously doesn’t have any of the quantitative data that the OASIS reports would have. If we did away with it, we’d probably get better feedback, more helpful, meaningful feedback.” (Administrator 3)*

Faculty reported that focus groups captured themes similar to those in feedback collected via online forms, which they interpreted as evidence of the small group’s ability to solicit representative feedback. There was also an emphasis on the importance of capturing different types of learners. Specifically, faculty hoped to see variability in how students engaged with the material (i.e., attending a live in-person lecture, watching a live remote lecture, or watching a recorded lecture) as well as differences in their preferred learning strategies.

*“They are largely repeating themes, seen, heard, both at Course Reps and the course evals. So, we have two sources of information that largely agree…” (Administrator 1)*

*“I didn’t notice anything that was very discrepant.” (Course Director 1)*

As students noted, faculty also mentioned that the lack of granular detail regarding individual lectures and small groups did not prevent the focus group from capturing the main points. A focus group allowed for the components that worked well or needed improvement to be highlighted without commenting on average-performing curricular elements. However, faculty expected to receive feedback about each component of the course.

*“Probably the big issues are getting caught, and they’re certainly getting caught in a better way with the focus group than they are with the OASIS signals.” (Course Director 3)*

*“By definition, it’s not as comprehensive, right? Because it doesn’t ask about each individual lecture. It’s more kind of a heuristic kind of thing, as opposed to a point by point addressing of small groups, each individual lecture. So, things that are forgotten or easily forgotten may get overlooked or not mentioned.” (Course Director 2)*

#### Engagement

Faculty were primarily concerned with student engagement in the focus groups as measured by attendance. Sample size was a concern for two primary reasons. First, despite the declining response rates to online forms, they captured approximately half of the class, while focus groups were intentionally limited to 10 students each. Many course directors relied on the larger sample size to justify initiatives and curricular changes and were uncomfortable with the decrease in numbers. Second, there was a concern about attendance. Since participation was not enforced, many chose not to participate. As a result, faculty were concerned about the bias introduced by a small group of students who elected to participate.

*“The fact that it’s ten students who are being queried on this [is a major concern]. It strikes me that ten is too small a number of students just because randomly you could get ten students who are outliers in one way or another.” (Course Director 2)*

#### Collaboration

Faculty repeatedly highlighted that collaboration between faculty and students is required to advance an educational initiative. Many saw focus groups as an opportunity to ask students specific questions about ideas for improvement, initiatives, and feedback received in previous years. Many believed that having an active dialogue between facilitators and faculty would further increase the utility of the focus groups and the quality of the feedback produced in the reports.

*“Where I feel like it would be particularly useful is if a course is having an issue or a problem or you need more information to figure out a direction to go. I feel like in those circumstances, the focus groups would serve a very important purpose.” (Administrator 2)*

*“If it’s a course that is struggling or it’s declining, then I think a focus group would be beneficial.” (Administrator 2)*

#### Relationship

Faculty expressed appreciation for the commitment of students and facilitators to soliciting high-quality feedback to improve the educational experience at the school. Importantly, faculty themselves were committed to curricular assessment, allowing innovative pilot programs, such as the focus groups, to take place.

*“I do support trying to figure out a way to continue to keep our finger on the pulse in a way that helps us make changes.” (Course Director 5)*

## Discussion

After completing interviews with focus group participants and faculty who received focus group reports, we identified five themes describing stakeholders’ experiences with and perceptions of a student-led focus group-based approach to curricular assessment: practices, environment, relationship, engagement, and collaboration. Compared with faculty, students emphasized the environment and the importance of a safe space to a much greater degree. However, both faculty and students prioritized practices and emphasized the importance of high-quality, representative feedback and a minimal administrative burden, respectively, achieved by the focus groups. Faculty members were concerned about the low sample size and attendance of focus groups.

Educational alliance theory was not used to guide data collection or coding, but it emerged as a useful interpretive lens during the later stages of analysis. Although educational alliance theory was originally developed to describe dyadic, interpersonal feedback relationships between learners and educators, several of the themes identified in this study resonate with constructs described in that literature [9]. In this context, educational alliance is best understood as an interpretive lens rather than a framework that guided study design or analysis. Our findings suggest that similar relational principles may be perceived by stakeholders in curricular feedback processes, though further theoretical work is needed to clarify how these concepts operate within institutional assessment systems.

We identified two competing components in curricular assessment. The first is sample size; faculty are most comfortable with the large sample sizes obtained through online forms. The higher participation rate is perceived as more representative of the consensus viewpoint and as preventing fringe opinions from having an overly prominent voice. The second essential component is quality. Faculty expect feedback to be not only representative but also accurate and actionable. To achieve this, feedback collection mechanisms must incentivize thoughtful student contributions.

Based on our study, most interviewed students either did not participate thoughtfully or did not complete the existing online evaluation forms used for curricular assessment. As such, neither sample size nor quality are optimized in the existing system. Focus groups, on the other hand, are intentionally limited in size to ensure a diversity of perspectives while still allowing each individual voice to be heard [16]. Despite the smaller sample size, faculty perceived the focus group reports to be comparable in thematic content to the reports generated by the online forms. Faculty further perceived that focus groups complemented existing evaluation mechanisms by providing additional content, facilitating discussion, and generating more actionable feedback. These stakeholder perceptions challenge the assumption that large sample sizes necessarily produce more useful curricular feedback. Institutions should consider whether supplementing high-volume evaluation methods with smaller, discussion-based approaches may improve the depth and usefulness of curricular feedback.

We are not alone in this assessment. Existing literature suggests that focus groups used for curricular assessment surpass online evaluation systems because they allow for open discussion among students, provide comprehensive reviews, and can highlight the class’s opinion rather than amplify the voice of one student [8]. Brandl et al. found that online evaluation forms captured only 49% of the recommendations obtained from a student-run focus group [7]. Furthermore, focus groups produced actionable feedback that led to changes in the preclerkship curriculum [7,8]. Focus groups have also been fruitful in the development and assessment of new curricula [17,18]. This study further contributes to the growing body of literature demonstrating the utility of focus groups in medical school curricular development and assessment.

After completing the focus group pilot at our institution, several areas for improvement were identified. First, future implementation efforts must consider the importance of attendance and mechanisms to either incentivize or enforce participation. Requiring attendance may be a reasonable approach to ensure the sample size remains 8–10 students per focus group; as participants point out, a small sample size may result in outlier rather than representative opinions despite random selection. Second, focus groups provide an opportunity for partnership between facilitators and faculty. As suggested by several faculty interviewees, focus groups could be used to address specific questions or concerns raised by course directors. For instance, new initiatives, proposed changes, or clarifications regarding previous feedback could be discussed productively as a group. Lastly, additional training for student facilitators may result in more actionable feedback rather than simply a summary of discussion points.

This study has limitations inherent to its design. First, participation in both the focus groups and subsequent interviews was voluntary, raising the possibility of self-selection bias and overrepresentation of individuals with particularly strong views. Although we achieved saturation, not all perspectives may have been captured. Second, although this study adopts an interpretive approach to understanding stakeholder experiences, certain analytic conventions (e.g., reporting relative emphasis of themes and use of inter-coder agreement) reflect pragmatic choices made to support rigor and clarity for evaluative audiences and should not be interpreted as claims of objectivity or generalizability. Third, while a neutral stance was maintained during interviews, the research team’s involvement in the initiative and commitment to curricular improvement may have introduced implicit biases that influenced data collection or interpretation. Fourth, this study focused on stakeholder perceptions of focus group-based feedback rather than a systematic comparison of feedback generated by focus groups and traditional surveys. Accordingly, perceived similarities between the two approaches reflect stakeholder impressions rather than an empirical comparison of feedback content. Future work should include formal comparative analyses of feedback content, actionability, and representativeness across evaluation methods. Fifth, this study took place at a single US medical school. For broader impact to be evaluated, replicability must be assessed at a number of diverse, international medical schools. Lastly, the findings primarily reflect early perceptions and may be influenced by novelty effects, since the interviews were conducted following an initial pilot. The sustainability of engagement with focus group–based feedback mechanisms remains an open question and warrants longitudinal evaluation.

This study examined stakeholder perceptions of an institutional feedback mechanism rather than interpersonal feedback relationships; therefore, any alignment with relational theories, such as educational alliance, should be interpreted with caution and does not imply direct application of those theories at the system level. Although several identified themes align with constructs described in educational alliance theory, this study did not aim to test, apply, or extend that theory. Rather, educational alliance was identified post hoc and used as an interpretive lens to contextualize stakeholders’ experiences at the systems level.

## Conclusion

Focus groups can be used as a supplemental tool for curricular assessment, complementing existing evaluation systems. Students appreciated the safe environment in which they could provide thoughtful, constructive feedback without a substantial time or workload burden. Faculty noted that feedback collected in this manner was similar in content to previous methods but generated additional context and, in some cases, offered actionable feedback that was lacking previously. However, concerns regarding sample size and attendance must be addressed prior to implementation. Given the challenges of curricular assessment, institutions should continue refining complementary evaluation approaches that combine the breadth of traditional survey methods with the depth and contextual understanding afforded by focus groups.

## Additional File

The additional file for this article can be found as follows:

10.5334/pme.1786.s1 Supplement. Supplementary Tables 1 and 2.
