# Supplementary material for: Beyond Online Course Evaluations: Student-Led Focus Groups for Curricular Feedback
Source: Perspect Med Educ. 2026 Sep 9;15(1):748–58. doi: 10.5334/pme.1786 (PMC13564938; doi:10.5334/pme.1786)
Supplement: Supplementary file 1 [file pme-15-1-1786-s1.pdf]

SUPPLEMENT

Supplementary Table 1: Questions utilized during interviews with participants.

Interviewee	Question
Students	Tell me about your experience participating in the focus group on XX/XX/XXXX date.
	Do you believe focus groups can capture a representative sample for feedback about a course? Why or why not?
	Did you feel that multiple opinions were represented?
	Was the focus group environment a safe space to share your opinion? What made it so?
	What could be done to make the focus groups better?
	What other changes would you recommend for the focus groups?
	Do you believe the focus groups can replace the previous curricular assessment mechanism of Oasis forms? Why or why not?
	Do you prefer participating in focus groups or Oasis?
	How do you envision course directors responding to the findings of the focus group?
	Do you believe there is benefit for participants of the focus group to review the summary generated by the facilitator? Why or why not?
	Is there anything else you would like to share about your experience during the focus group?
Faculty	Tell me about your experience receiving the focus group report?
	Do you believe focus groups can capture a representative sample for feedback about a course? Why or why not?
	Did you feel that multiple opinions were represented?
	Did the focus group report provide necessary context for feedback?
	Did the focus group report provide actionable recommendations?
	What changes could be made to the course based on the information provided in the focus group report?
	Do you believe the focus groups can replace the previous curricular assessment mechanism of Oasis forms? Why or why not?
	Do you prefer the report generated by focus groups or Oasis?
	What changes would you recommend for the focus groups?
	Is there anything else you would like to share about your experience with the focus group?
Administrators	Tell me about your experience receiving focus group reports?
	What are some of the benefits and drawback of focus group that you see?
	Do you believe the data in the focus group report is reliable? Why or why not?
	How do the findings from the focus groups compare to the findings of Oasis reports?
	What role do you believe focus group reports should play in curricular assessment?
	Is there anything else you would like to share about your experience with the focus group?

Supplementary Table 2: Themes and associated definitions identified following interviews with students, faculty, and administrators.

Code	Definition
Collaboration	Active, intentional partnership between various stakeholders *students, teachers, administrators) to achieve common educational goals.
Co-creation	Stakeholders work together to co-create meaningful experiences, ensuring the needs and interests of all stakeholders are addressing. This includes faculty input in focus group use and content.
Shared responsibility	All stakeholders are responsible for participating in planning, implementing, and assessing the educational initiative
Engagement	Active involvement of all stakeholders in the educational initiative (focus groups for curricular assessment).
Authentic interest	Stakeholders should be invested in the success of the initiative.
Honesty	Stakeholders should be honest when participating and assessing the initiative.
Sample size	Students must show investment and interest in the process by attending focus groups. Poor attendance may indicate that students are not active participants in the initiative.
Environment	Includes both the physical space and the context in which the initiative is attempted.
Safety	Stakeholders should feel safe to participate in the environment. This includes

	physical, psychological, cultural, and social safety.
Anonymous	To the extent this contributes to the safety of the environment.
Peer participants	To the extent this contributes to the safety of the environment.
Facilitators	To the extent this contributes to the safety of the environment.
Food	To the extent this contributes to the safety of the environment.
Intimate setting	To the extent this contributes to the safety of the environment.
No faculty	To the extent this contributes to the safety of the environment.
Practices	Shared educational methods, strategies, and behaviors that guide how stakeholders interact with the initiative and with each other.
Alignment with expectations and needs	All practices should align with the expectations and needs of all stakeholders. This code includes alignment and misalignment. This code also refers to the utility of focus groups or Oasis forms to meet evaluation needs and use cases.
Longitudinal feedback	Feedback should cover the duration of a course and be reproducible year to year.
Quality of feedback	Feedback should be actionable, have an appropriate level of detail, etc.
Representative feedback	Feedback should be representative of the student body's view.
Focus groups	Comment on whether the focus groups solicit representative feedback.
Oasis	Comment on whether Oasis online forms solicit representative feedback.
Thoughtful engagement with feedback	Feedback should be completed in a thoughtful manner.
Student engagement with Oasis	Comment on whether Oasis online forms solicit thoughtful feedback.
Time or work burden	Comment regarding the time or work burdens of Oasis online forms.
Student participation with Oasis	Comment on whether students complete Oasis online forms.
Student engagement in focus groups	Comment on whether focus groups solicit thoughtful feedback.
Content delivery	Recognition that perspectives and feedback may vary depending on the mechanism for content delivery (live, recorded, a/synchronous, etc.).
Relationship	Refers to the trust, communication, and mutual respect that connects the different stakeholders in the learning environment.
Commitment	Sustained dedication and long-term investment in the success of the initiative.
Respect	The recognition of the value, perspectives, and contributions of stakeholders.
Trust	The confidence and belief in the integrity, competence, and reliability of all involved stakeholders and/or the initiative itself.
Closed-loop communication	Belief that closed-loop communication is important between stakeholders.
Course Representative	Stakeholders have trust in course representatives to represent the views of students.
Faculty	Students trust faculty to act on feedback provided through focus groups and online forms.
